# Impacts of Fructose on Intestinal Barrier Function, Inflammation and Microbiota in a Piglet Model

**DOI:** 10.3390/nu13103515

**Published:** 2021-10-06

**Authors:** Pingting Guo, Haichao Wang, Linbao Ji, Peixia Song, Xi Ma

**Affiliations:** 1 State Key Laboratory of Animal Nutrition, Department of Animal Nutrition and Feed Science, College of Animal Science and Technology, China Agricultural University, Beijing 100193, China; ptguo@cau.edu.cn (P.G.); jilinbao0126@cau.edu.cn (L.J.); px.songlab@gmail.com (P.S.); 2 Department of Animal Science, College of Animal Science (College of Bee Science), Fujian Agriculture and Forestry University, Fuzhou 250003, China; 3 Department of Animal Science, College of Life Sciences and Food Engineering, Hebei University of Engineering, Handan 056038, China; qiusuoba@163.com

**Keywords:** fructose, intestinal barrier, microbes, weaned piglets

## Abstract

The metabolic disorder caused by excessive fructose intake was reported extensively and often accompanied by intestinal barrier dysfunction. And the rising dietary fructose was consumed at an early age of human. However, related researches were almost conducted in rodent models, while in the anatomy and physiology of gastrointestinal tract, pig is more similar to human beings than rodents. Hence, weaned piglets were chosen as the model animals in our study to investigate the fructose’s impacts on intestinal tight junction, inflammation response and microbiota structure of piglets. Herein, growth performance, inflammatory response, oxidation resistance and ileal and colonic microbiota of piglet were detected after 35-day fructose supplementation. Our results showed decreased tight junction gene expressions in piglets after fructose addition, with no obvious changes in the growth performance, antioxidant resistance and inflammatory response. Moreover, fructose supplementation differently modified the microbiota structures in ileum and colon. In ileum, the proportions of Streptococcus and Faecalibacterium were higher in Fru group (fructose supplementation). In colon, the proportions of Blautia and Clostridium sensu stricto 1 were higher in Fru group. All the results suggested that tight junction dysfunction might be an earlier fructose-induced event than inflammatory response and oxidant stress and that altered microbes in ileum and colon might be the potential candidates to alleviate fructose-induced intestinal permeability alteration.

## 1. Introduction

The fructose consumption worldwide has reached a historic high level. And the children have experienced an overall rise in the amount of dietary fructose consumption in the United States over the past two decades [1]. Excessive fructose intake can induce sever fatty liver disease, obese, diabetes, metabolic syndrome and so on [2,3,4]. Previously, it was deemed that the carbotoxicity of fructose in body health resulted from the wild and uncontrolled fructose metabolism in liver. However, a recent study pointed out that 90% dietary fructose was converted into glucose and lactate by the small intestine before it reached liver [5]. Only excess fructose spilled over to liver and colonic microbiota when fructose intake exceeds the maximal clearance capacity of the small intestine [5]. Hence, the significance of the small intestine on fructose metabolism have been increasingly emerging. Meanwhile, the dysfunction of intestinal barrier was frequently presented in the above-mentioned metabolic diseases caused by excess fructose intake [6,7,8]. A recent study conducted in mice also showed that high-fructose diet increased gut permeability due to alterations to the tight junction proteins although body weight didn’t change [9]. Tight junction is the predominant barrier that prevents luminal large molecules and detrimental microbial metabolites from invading internal environment. Its dysfunction is the early event of cell apoptosis and reflects the early damage of cells [10]. It was reported that the intestinal tight junction dysfunction might result from oxidative stress, activated immunologic response along with high-level inflammation factors such as IL-1β, TNF-α and INF-γ, disordered intestinal microbiota and/or other stress [11,12,13]. For example, IL-1β caused a rapid activation of mitogen-activated protein kinase kinase kinase 1, which activated the canonical pathway of nuclear factor kappa-B to induce myosin light chain kinase (MLCK) activation, then resulting in the molecular reorganization of tight junction structure and composition and larger intestinal permeability [14]. However, most studies about the impacts of fructose on intestinal health were conducted with rodent model, whereas humans have a smaller intestinal surface area than rodents [15]. Compared with rodents, the gastrointestinal tract anatomy and physiology of humans is more similar to pig [16]. Meanwhile, the high-fructose consumption has developed from children stage [1]. Therefore, this study attempted to explore the effects of fructose on intestinal tight junction in terms of oxidative stress, inflammatory response and intestinal microbiota by using a weaned piglet model.

## 2. Materials and Methods

### 2.1. Piglets Feeding Experiment

All experimental protocols were carried out with approval of the China Agricultural University Animal Care and Use Committee (CAU20201247-1). A total of 60 weaned piglets (Duroc × Landrace × Yorkshire) with an initial body weight of 6.52 ± 0.37 kg were randomly assigned to 2 treatments (6 replicate pens per treatment and 5 piglets per pen). Piglets were fed with either basal diet (Con) or 0.2% fructose-supplemented diet (Fru). The dosage of fructose used in this study was referred to previous reports [17,18,19]. The basal diet was formulated according to National Research Council recommendations (Table 1). The fructose (purity: 99.7%) used in our study was supplied from Shandong XiWang Biotechnology Co., Ltd. (Shandong, China).

The trial lasted for 35 days. The body weight of each piglet and the feed intake per pen were recorded on day 0, day 14 and day 35. The daily gain (ADG), daily feed intake (ADFI) and F/G (feed/gain) were calculated. On day 35, one pig in each pen was randomly selected and sacrificed for sampling after fasting overnight. The blood samples and ileal segments were collected for immune factors and antioxidant indices analyses. Ileal segments were also collected and stored at −80 °C for Quantitative Real-Time PCR (RT-PCR) analyses of gut tight junction genes. Ileal and colonic digesta were gathered, immediately snap-frozen using liquid nitrogen, and stored at −80 °C for 16S rDNA sequencing.

All piglets were supplied by the Swine Nutrition Research Center of the National Feed Engineering Technology Research Center (Chengde, Hebei Province, China). Piglets were housed in nursery room and allowed free access to diets and water ad libitum. Relative humidity was maintained at 65–75% and temperature was controlled at 22 °C–26 °C.

### 2.2. Quantitative RT-PCR

Total RNA extraction, reverse transcription and quantitative RT-PCR analysis were conducted as described previously [20]. Briefly, total RNA was obtained using TRIzol reagent and reverse transcription was performed with the ThermoSCRIPT RT-PCR System according to the manufacturer’s protocol (Invitrogen Life Technologies, Carlsbad, CA, USA). Quantitative real-time PCR analyses of ZO1, OCLN, CLDN1 and MLCK were carried out using the DNA double-strand specific SYBR Green I dye (Roche, Basel, Switzerland) and the TaqMan Sequence Detection System. The gene-specific primers were shown in Table 2. ACTB was chosen as the reference gene.

### 2.3. Detections of Antioxidant Indices and Immune Factors

The antioxidant indices and immune factors of serum and ileum were determined using corresponding assay kits according to the manufacturer’s instructions. Except the assay kit of complement component 3 (C3) supplied from Sanwei Bioengineering Group Co., Ltd., other kits were purchased from Nanjing Jiancheng Bioengineering Institute.

The brief detection process was present here. The blood samples were centrifuged at 3000× *g* for 10 min at room temperature and the resultant supernatants were obtained as serum samples. Ileal segments were minced and homogenized (10% *w*/*v*) in ice-cold sodium-potassium phosphate buffer (0.01 M, pH 7.4) containing 0.86% NaCl. The homogenate was centrifuged at 3000× *g* for 10 min at 4 °C. The resultant supernatants and serum samples were analyzed for the activities of glutathione peroxidase (GSH-Px), superoxide dismutase (SOD) and the contents of malondialdehyde (MDA), glutathione (GSH), and immune factors C3, IL-1β, IL-2, IFN-γ and TNF-α via ELISA. The total protein concentration of the supernatants was detected using the BCA protein assay kit (Pierce, Rockford, IL, USA). All absorbance levels were measured using a Synergy4 Multifunction Microplate Reader (Bio-Tek Instruments, Winooski, VT, USA).

### 2.4. 16S rDNA Sequencing and Data Analysis

The procedure of 16S rDNA sequencing was described in detail in our previous study [21]. The simplified workflow was present here. The bacterial DNA of ileal and colonic digesta were extracted by using Stool DNA Kit (D4015-01, Omega Bio-tek, Norcross, GA, USA), then amplified with the primers of V3-V4 region in bacterial 16S rDNA. The amplicons were purified and then sequenced via the Illumina MiSeq platform. After quality control and assembly, the sequencing data were used for analyses of microbiota α diversity (Chao index and Shannon index), β diversity (PCoA and ANOSIM analyses), and variation between groups at different species levels, spearman correlation analysis and Tax4Fun functional predictions. All raw sequencing data have been deposited in the NCBI Sequence Read Archive under the BioProject PRJNA683707.

### 2.5. Statistical Analyses

Statistical analysis was performed using GraphPad Prism Version 9.0 if not stated otherwise in the methods section. Microbial composition differences between groups at different species levels were calculated using Wilcoxon rank-sum test. Differences of Tax4Fun functional predictions between groups were calculated using Multiple Unpaired t Test and statistical significance was assumed at FDR (False Discovery Rate) ≤ 0.01. Beyond above analyses, other differences between groups were calculated using unpaired Student’s t tests. Statistical significance was assumed at *p* ≤ 0.05. In figures, significances were annotated with the following markers: *, *p* ≤ 0.05; **, *p* ≤ 0.01; ***, *p* ≤ 0.001; unless stated otherwise, data were reported as means ± SEM.

## 3. Results

### 3.1. The Effect of Fructose on Growth Performance in Weaned Piglets

As shown in Table 3, compared with Con group, no obvious changes were observed in growth performance of weaned piglets in Fru group during the whole trial period. There was only a decreasing tendency on F/G at first two weeks in Fru group (*p* = 0.076). 

### 3.2. The Effect of Fructose on Ileal Tight Junction Gene Expressions in Weaned Piglets

**Figure 1:**
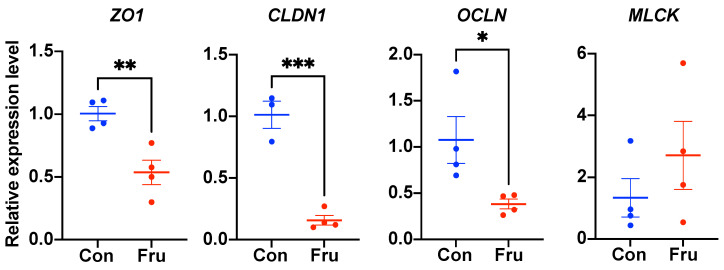
The ileal barrier function of weaned piglets fed a basic or 0.2% fructose-supplemented diet. The relative mRNA expression levels of tight junction (ZO1, CLDN1, OCLN) and MLCK genes were shown here; *n* = 4. Con, the control group; Fru, the 0.2% frucose-supplemented group. *, *p* ≤ 0.05; **, *p* ≤ 0.01; ***, *p* ≤ 0.001.

As demonstrated in Figure 1, the expression levels of tight junction genes ZO1, CLDN1 and OCLN were obviously down-regulated (*p* ≤ 0.05) while the MLCK mRNA level wasn’t affected by the fructose addition.

### 3.3. The Effects of Fructose on Serous and Ileal Immunologic Function and Oxidation Resistance in Weaned Piglets

The statistical analyses of immune factors and antioxidant indices in serum and ileum were displayed in Table 4 and Table 5 respectively. The contents of MDA and GSH and the activities of SOD and GSH-Px weren’t affected by 0.2% fructose supplementation. Meanwhile, no obvious changes were observed in the serous and ileal contents of C3, IFN-γ and TNF-α. There was only an increasing tendency in the contents of serous IL-2 and ileal IL-1β after fructose supplementation (*p* = 0.052 and *p* = 0.094, respectively).

### 3.4. The Effect of Fructose on Ileal Microbiota Structure of Weaned Piglets

**Figure 2:**
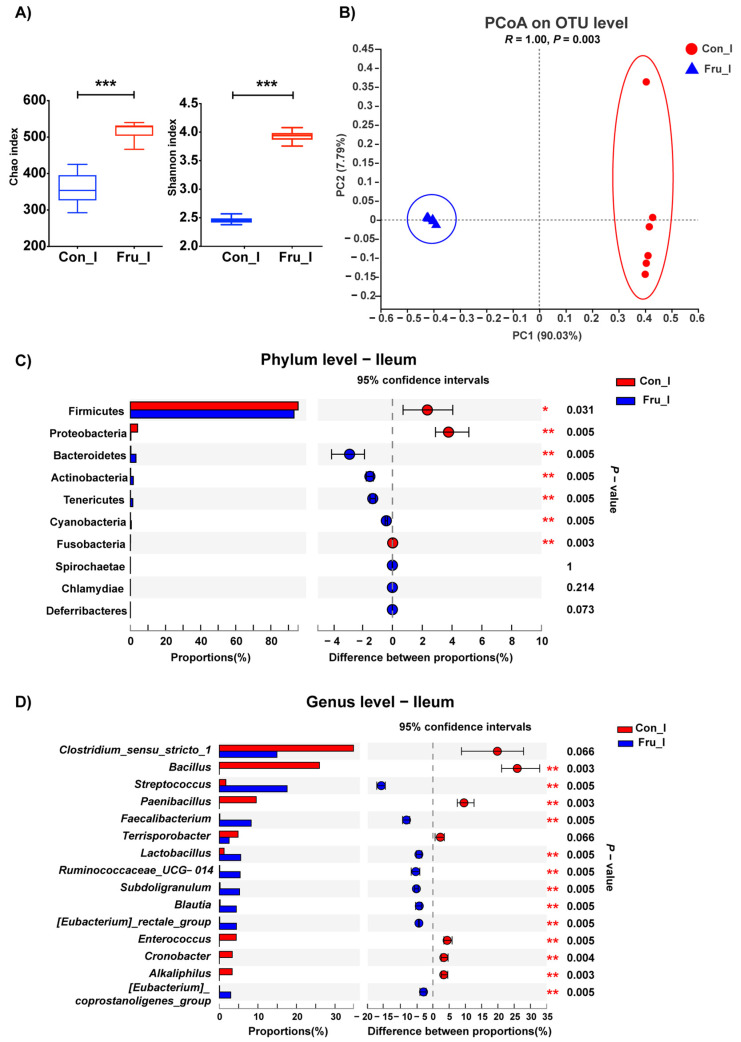
The microflora composition in ileal digesta of weaned piglets fed a Con or 0.2% fructose-supplemented diet. The Chao and Shannon indexes (**A**), the PCoA and ANOSIM analyses on OTU level (**B**) and the relative abundance of bacteria at Phylum (**C**) and Genus (top15) (**D**) levels were shown here; *n* = 6. Ilea in Con group and Fru group were represented as Con_I and Fru_I respectively. *, *p* ≤ 0.05; **, *p* ≤ 0.01; ***, *p* ≤ 0.001.

The α diversity indexes of bacteria in ileal digesta were presented in Figure 2A. Chao and Shannon indexes were higher in Fru group (*p* ≤ 0.05). The PCoA and ANOSIM analyses on OTU level showed that the bacterial composition between Con and Fru groups were significantly discrepant (*p* = 0.003) (Figure 2B). At Phylum level (Figure 2C), Firmicutes were the predominant bacteria in ileal digesta in both groups, with the proportion of 95.6% and 93.2% in Con group and Fru group respectively. The proportions of Firmicutes and Proteobacteria decreased, but the proportions of Bacteroidetes, Actinobacteria and Tenericutes increased with the fructose supplementation (*p* ≤ 0.05). At Genus level (Figure 2D), the proportions of Streptococcus and Faecalibacterium were remarkably higher, while the proportions of Bacillus, Paecalibacterium, Enterococcus, Cronobacter and Alkaliphilus were lower in Fru group (*p* ≤ 0.01). 

**Figure 3:**
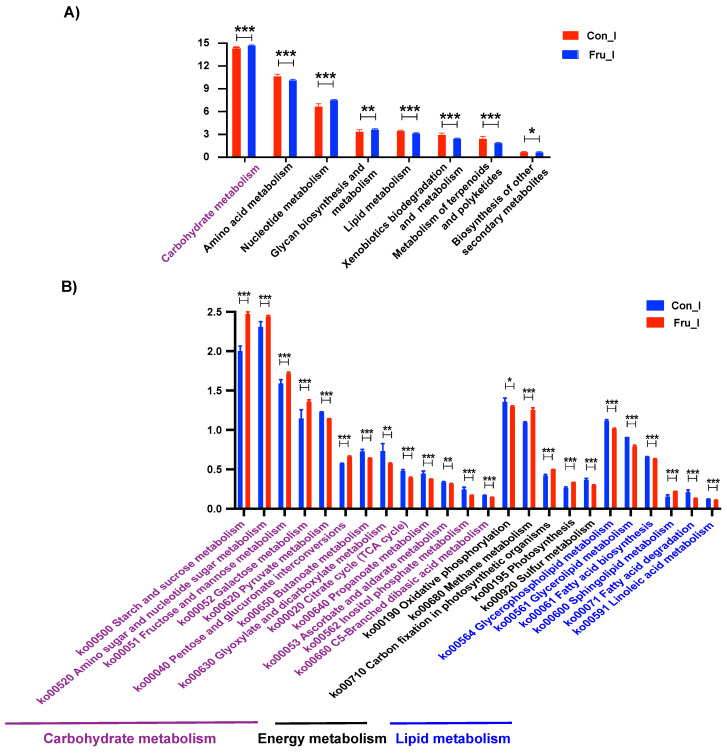
Tax4Fun functional predictions of ileal microbiota. The predicted differential metabolism pathways at Pathway Level 2 (**A**) and the predicted differential carbohydrate, energy and lipid metabolism pathways at Pathway Level 3 (**B**) were shown here. Data were reported as means ± SD, *n* = 6. Ilea in Con group and Fru group were represented as Con_I and Fru_I respectively. *, *p* ≤ 0.05; **, *p* ≤ 0.01; ***, *p* ≤ 0.001.

Meanwhile, Tax4Fun functional prediction was conducted to reveal the alteration of bacterial metabolic pattern in ileal digesta due to fructose addition (Figure 3). Eight metabolism pathways at Pathway Level 2 were modified and carbohydrate metabolism was more robust in Fru group (FDR ≤ 0.01) (Figure 3A). Herein, fructose supplementation enhanced the metabolisms of most saccharides such as starch, sucrose and fructose and weakened the metabolisms of various lipids (FDR ≤ 0.01) (Figure 3B).

### 3.5. The Effect of Fructose on Colonic Microbiota Structure of Weaned Piglets

**Figure 4:**
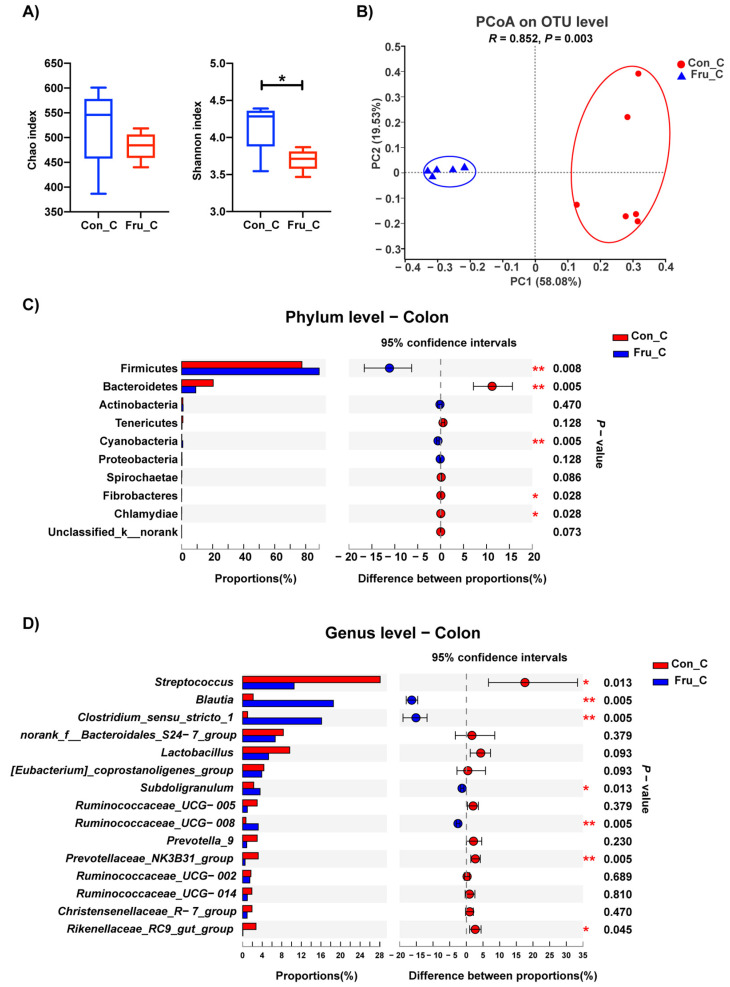
The microflora composition in colonic digesta of weaned piglets fed a Con or 0.2% fructose-supplemented diet. The Chao and Shannon indexes (**A**), the PCoA and ANOSIM analyses on OTU level (**B**) and the relative abundance of bacteria at Phylum (**C**) and Genus (top 15) (**D**) levels were shown here; *n* = 6. Colons in Con group and Fru group were represented as Con_C and Fru_C respectively. *, *p* ≤ 0.05; **, *p* ≤ 0.01.

The modifications of colonic microbiota composition after fructose supplementation were displayed in Figure 4. Shannon index, an α diversity index, was lower in Fru group (*p* ≤ 0.05) (Figure 4A). Meanwhile, the microbiota composition between Con and Fru groups was obviously discrepant according to the PCoA and ANOSIM analyses on OTU level (*p* = 0.003) (Figure 4B). At Phylum level (Figure 4C), the proportion of Firmicutes was increased (from 77.5% to 88.7%), while the proportion of Bacteroidetes was decreased (from 20.3% to 9.1%) with the fructose supplementation (*p* ≤ 0.01). At Genus level (Figure 4D), the proportions of Blautia and Clostridium sensu stricto 1 were higher while the proportion of Streptococcus was lower in Fru group (*p* ≤ 0.05).

## 4. Discussion

Increasing high-fructose corn syrup consumption accelerated the development of metabolic diseases nowadays and came up at a very young age of human. The small intestine, as the most important organ that absorb and metabolize fructose, exert a noticeable influence on consequent metabolic disorder after excessive fructose intake. The intestinal tight junction dysfunction was frequently observed when high-fructose diets were provided to rodent models in many studies [7,8,9]. However, rodents intestinal anatomy similarity to human is inferior to pigs. So, we tried to revalidate this phenomenon with a piglet model in this study. 

The doses of fructose given to piglets was 2.5 g/d by Kidder et al. [17] and 2.0 g/day by Aherne et al. [18], respectively. Bird and Hartmann viewed that the dose of 2.5 g/day fructose represented an unusually high intake of simple carbohydrate for sucking piglets [19]. The highest average daily feed intake of piglets was about 1000 g/day, so we supplemented 0.2% of fructose and the piglets could ingest 2.0 g fructose one day. In this study, the growth performance of piglets was not affected. Several researches demonstrated an increased weight gain because of high-fructose diets [22,23], while other study found no changes in body weight of mice [9]. Circulatory fructose could stimulate endogenous glucose production and lipid synthesis, which can increase fasted and postprandial glucose and triglyceride concentrations, then contributing to weight gain [24]. The different results shown here might be caused by the discrepant dose, model animal type or trial period in these studies.

Tight junction is the vital component of intestinal physical barrier. In our study, the ileal mRNA expression levels of three main tight junction genes, ZO1, OCLN and CLDN1 decreased with fructose supplementation. The tight junction damage induced by high-fructose was also discovered in many previous works [9,12,25,26]. Tight junction proteins constitute the dynamic structures of paracellular pathway, which is responsible for passive unregulated passage of water, electrolytes and small molecules [27]. Impairment of tight junction could result in looser intercellular connection, larger intestinal permeability and even epithelial cell abscission, followed by epithelial layer invasion of luminal large molecules and activation of inflammatory response. MLCK was a vital protein to phosphate myosin light chain, contract perijunctional actomyosin ring, promote tight junction proteins endocytosis and then induce larger intestinal permeability [28,29,30]. In the current study, there was no significant change in the mRNA expression of MLCK between two groups.

According to the previous reports, the integrity and dynamic changes of tight junction could be affected by various kinds of stress such as oxidative stress, inflammatory response and unbalanced intestinal microbiota [11,12,13,31]. Hence, we further detected the alterations of above phenomena. To our surprise, there was no obvious change on antioxidant molecules in serum and ileum, which was inconsistent with some existing researches [2,32,33]. It was reported that fructose drinking caused increased cytochrome P450-2E1, inducible nitric oxide synthase, and nitrated proteins in small intestine and liver of rodents. Then the intestinal tight junction proteins were nitrated and ubiquitinated, resulting in increased intestinal permeability [2]. Reinforced lipid peroxidation and protein carbonylation were also found after fructose addition [33].

Similar to oxidative stress, the inflammatory response also wasn’t altered by fructose, only to find an increasing tendency of serous IL-2 and ileal IL-1β. However, the serous contents of IL-1β, IL-6, TNF-α and bacterial endotoxin were reported to increase due to high-fructose treatment [2,34,35,36]. And in a recent study, the high-fructose diet was found to decrease the protein expressions of ZO1 and occludin, along with increased expression of the tight junction-disrupting cytokines TNF-α and IL-1 β in the colon of mice [9]. Hence, no obvious oxidative stress and inflammatory response found in our study might result from our short trial period (only 5 weeks). Taken together with the decreased expression levels of intestinal tight junction genes, we supposed that tight junction dysfunction might be the early event, along with oxidative stress and inflammatory response as its consequent effects, after high-fructose treatment.

As an important part of regulating metabolism, gut microbiota plays an important role in trophic decomposition and absorption. Numerous studies have shown a high correlation between intestinal microbiota and gut barrier function [37,38]. Moreover, intestinal microbiota composition and structure may vary due to the difference in the location and physiological function of different intestinal segments [39]. In our study, the intestinal microbiota structures in ileum and colon were modified by high-fructose supplementation and the modifications in ileum and colon were obviously discrepant.

In ileum, fructose fermentation was promoted according to the results of functional prediction, and the abundance and diversity of ileal microbiota were enriched after fructose treatment. The proportions of aerobic Bacillus and Paenibacillus dropped off, along with elevated anaerobic Streptococcus and Faecalibacterium, which do lactate fermentation and butyrate fermentation respectively. The results indicated that fructose supplementation inhibited bacterial aerobic respiration in ileum, which might indirectly suppress the expressions of tight junction genes. Our results were inconsistent with an earlier report, which demonstrated that more abundant Faecalibacterium, less zonulin, whose expression level was positively correlated with intestinal permeability [40]. Moreover, short-chain fatty acids (SCFAs), the fermentation products of Faecalibacterium, were of benefit to intestinal barrier function [25,41]. Hence, further intensive studies are required to reveal the underlying cause leading to opposite results herein.

Unlike ileum, the bacterial diversity in colon was weakened. The result is similar with a study conducted in mice [9]. The proportions of Blautia and Clostridium sensu stricto 1 increased while the proportion of Streptococcus dropped off. Blautia species are one of the most abundant members in gastrointestinal tract [42]. Besides, Blautia is responsible for acetogenesis by utilizing hydrogen and carbon dioxide [43]. Acetogenesis is of great interest for animal and human health by decreasing the total gas volume in the colon and providing energy source for colonic epithelial cells. Clostridium sensu stricto 1 is a butyrate-producer and was also found increased in cecum of rat after high-fructose treatment [34]. However, decreased Streptococcus is a lactate-producer. The results indicated that the SCFA fermentation were enhanced while lactate fermentation was weakened in colon with fructose supplementation. Besides SCFAs, lots of unknown factors are speculated to play a vital role in the complicated interaction between intestinal barrier and microbiota. And the altered microbes are the potential candidates to cope with the fructose-induced decrease of intestinal tight junction gene expressions.

## 5. Conclusions

Fructose supplementation in piglet diet obviously suppressed the expressions of ileal tight junction genes, along with modifications in ileal and colonic microbiota structures. Our results suggested that tight junction dysfunction might be an earlier fructose-induced event than inflammatory response and oxidant stress and that altered microbes in ileum and colon might be the potential candidates to alleviate fructose-induced intestinal permeability alteration.

## Figures and Tables

**Table 1:** The ingredient composition and nutrient levels of diets (%, as-fed basis).

Ingredients	Content (%)	Nutrient Levels	Content
Extruded maize meal	54.19	Gross energy (MJ/kg)	16.95
Dehulled soybean meal	20.70	Dry matter (%)	91.41
Extruded soybean	11.00	Crude protein (%)	20.26
Whey power	4.00	Ether extract (%)	8.11
Fish meal	3.00	Calcium (%)	0.87
Wheat bran	1.50	Total Phosphorus (%)	0.71
Dicalcium phosphate	2.20		
Glucose	1.00		
Limestone	0.80		
L-Lysine·HCl	0.35		
L-Threonine	0.18		
DL-Methionine	0.05		
Tryptophan	0.03		
Premix ^1^	1.00		
Total	100.00		

^1^ Premix supplied per kg diet: vitamin A, 9000 IU; vitamin D3, 3000 IU; vitamin E, 20 mg; vitamin K3, 3 mg; vitamin B12, 0.2 mg; niacin, 30 mg; pantothenic acid, 15.0 mg; choline chloride, 400 mg; Zn, 75 mg; Mn, 60 mg; Fe, 75 mg; Cu, 150 mg; I, 0.35 mg; Se, 0.30 mg.

**Table 2:** Primers used for quantitative RT-PCR.

Gene	Forward (5′-3′)	Reverse (5′-3′)
ACTB	ACACGGTGCCCATCTACGAG	GCTTCTCCTTGATGTCCCGC
ZO1	AGCCATCCACTCCTGCCTAT	GACGGGACCTGCTCATAACT
OCLN	CTTTCTCAGCCAGCGTATTC	AGGCAAGCGTGGAGGCAACA
CLDN1	CATTGCTATCTTTGCCTGTG	GCCATAACCGTAGCCATAAC
MLCK	CCTGTCCTGGTATGGCTCCT	CTGCGGCATGTGGCTAGTTC

**Table 3:** The effect of fructose on growth performance in weaned piglets.

Items	Con	Fru	*p*-Value
1–14 days			
ADG, g	224 ± 9	236 ± 12	0.439
ADFI, g	490 ± 20	471 ± 20	0.534
F/G	2.19 ± 0.05	2.01 ± 0.07	0.076
15–35 days			
ADG, g	479 ± 4	473 ± 16	0.694
ADFI, g	1064 ± 20	1043 ± 36	0.619
F/G	2.22 ± 0.03	2.21 ± 0.03	0.780
1–35 days			
ADG, g	377 ± 4	378 ± 11	0.947
ADFI, g	834 ± 19	814 ± 27	0.556
F/G	2.21 ± 0.03	2.15 ± 0.02	0.110

Data were shown as mean ± SEM (*n* = 6); Con: basic diet; Fru: basic diet with 0.2% fructose; ADG, average daily gain; ADFI, average daily feed intake; F/G, ADFI/ADG.

**Table 4:** The effect of fructose on serous and ileal immune factors in weaned piglets.

Items	Con	Fru	*p*-Value
**Serum**			
C3, g/L	0.92 ± 0.02	0.93 ± 0.03	0.738
IL-1β, ng/mL	0.21 ± 0.03	0.19 ± 0.02	0.762
IL-2, ng/mL	4.85 ± 0.30	6.86 ± 0.67	0.052
IFN-γ, pg/mL	46.31 ± 4.92	32.71 ± 5.74	0.146
TNF-α, ng/mL	1.03 ± 0.15	1.54 ± 0.31	0.214
**Ileum**			
C3, g/g TP	0.18 ± 0.01	0.20 ± 0.01	0.222
IL-1β, ng/mg TP	0.02 ± 0.00	0.03 ± 0.01	0.094
IL-2, ng/mg TP	0.55 ± 0.10	0.63 ± 0.08	0.548
IFN-γ, pg/mg TP	3.92 ± 0.54	4.43 ± 0.72	0.597
TNF-α, ng/mg TP	0.10 ± 0.01	0.07 ± 0.02	0.319

Values were shown as mean ± SEM (*n* = 6); Con: basic diet; Fru: basic diet with 0.2% fructose; C3, complement component 3; IL-1β, interleukin 1β; IL-2, interleukin 2; IFN-γ, Interferon-γ; TNF-α, tumor necrosis factor-α; TP, total protein.

**Table 5:** The effect of fructose on serous and ileal oxidation resistance in weaned piglets.

Items	Con	Fru	*p*-Value
**Serum**			
MDA, nM/mL	5.65 ± 0.24	5.60 ± 0.35	0.900
GSH, μM/L	3.76 ± 0.09	3.88 ± 0.18	0.573
SOD, U/mL	82.67 ± 5.83	69.45 ± 4.35	0.143
GSH-Px, U/mL	1010 ± 143	1086 ± 124	0.707
**Ileum**			
MDA, nM/mg TP	0.51 ± 0.07	0.67 ± 0.12	0.315
GSH, μM/mg TP	0.31 ± 0.03	0.40 ± 0.04	0.139
SOD, U/mg TP	8.07 ± 0.59	7.51 ± 0.28	0.422
GSH-Px, U/mg TP	66.88 ± 8.06	78.89 ± 11.76	0.432

Values were shown as mean ± SEM (*n* = 6); Con: basic diet; Fru: basic diet with 0.2% fructose; MDA, malonaldehyde; GSH, glutathione; SOD, superoxide dismutase; GSH-Px, glutathione peroxidase; TP, total protein.

## Data Availability

The raw data are available for readers if necessary.
